# Never the twain shall meet? - a comparison of implementation science and policy implementation research

**DOI:** 10.1186/1748-5908-8-63

**Published:** 2013-06-10

**Authors:** Per Nilsen, Christian Ståhl, Kerstin Roback, Paul Cairney

**Affiliations:** 1 Division of Healthcare Analysis, Department of Medical and Health Sciences, Linköping University, Linkoping, Sweden; 2 National Centre for Work and Rehabilitation, Department of Medical and Health Sciences, Linköping University, Linkoping, Sweden; 3 Division of History and Politics, University of Stirling, Stirling, Scotland

**Keywords:** Policy, Implementation, Top-down, Bottom-up, Determinants, Context, Interdisciplinarity

## Abstract

**Background:**

Many of society’s health problems require research-based knowledge acted on by healthcare practitioners together with implementation of political measures from governmental agencies. However, there has been limited knowledge exchange between implementation science and policy implementation research, which has been conducted since the early 1970s. Based on a narrative review of selective literature on implementation science and policy implementation research, the aim of this paper is to describe the characteristics of policy implementation research, analyze key similarities and differences between this field and implementation science, and discuss how knowledge assembled in policy implementation research could inform implementation science.

**Discussion:**

Following a brief overview of policy implementation research, several aspects of the two fields were described and compared: the purpose and origins of the research; the characteristics of the research; the development and use of theory; determinants of change (independent variables); and the impact of implementation (dependent variables). The comparative analysis showed that there are many similarities between the two fields, yet there are also profound differences. Still, important learning may be derived from several aspects of policy implementation research, including issues related to the influence of the context of implementation and the values and norms of the implementers (the healthcare practitioners) on implementation processes. Relevant research on various associated policy topics, including The Advocacy Coalition Framework, Governance Theory, and Institutional Theory, may also contribute to improved understanding of the difficulties of implementing evidence in healthcare. Implementation science is at a relatively early stage of development, and advancement of the field would benefit from accounting for knowledge beyond the parameters of the immediate implementation science literature.

**Summary:**

There are many common issues in policy implementation research and implementation science. Research in both fields deals with the challenges of translating intentions into desired changes. Important learning may be derived from several aspects of policy implementation research.

## Background

Advances in research have led to increased opportunities for better patient treatment and care, offering the potential for improved health and well-being of populations. However, numerous studies have documented that many patients do not obtain treatments with proven effectiveness, or receive care that is of little benefit or even harmful [1-4]. Healthcare researchers, practitioners, and policy makers have increasingly recognized the critical role of implementation science to reduce the gap between what has been shown in research to be effective and what is actually practiced in healthcare. Implementation science examines the ways in which healthcare practitioners can use research findings more effectively in routine clinical practice to develop a more research-informed practice.

Many of society’s health problems require research-based knowledge acted on by healthcare practitioners together with implementation of political measures from governmental agencies. A review of the ten most important public health achievements of the twentieth century in the United States showed that they were all influenced by public policies, such as seat belt laws or regulations governing permissible workplace exposures [5]. Thus, it is important to conduct research into the implementation of ‘Big P’ policies in the form of formal laws, rules and regulations alongside investigations into ‘small p’ healthcare policies such as guidelines and management decisions that can affect the use of research in clinical practice. Policy implementation research, the study of ‘how governments put policies into effect’ [6,7], has been conducted since the early 1970s.

There may be important parallels between implementation science and policy implementation research. In some cases, the implementation object may very well be the same in both fields, *e*.*g*., a guideline based on public health policies that prescribes the use of certain methods in healthcare. However, knowledge exchange and cross-fertilization between the two fields has been minimal. Research overviews and literature reviews in either field tend to mention few if any researchers or publications from the other field. Does this separation mean that there is little useful knowledge or experience that can be shared between the two fields? The aim of this paper is to describe the characteristics of policy implementation research, analyze some key similarities and differences and discuss how learning derived in four decades of policy implementation research might inform the younger field of implementation science.

To address the study aims, we undertook a narrative review of selective literature on implementation science and policy implementation research, predominantly relying on secondary data in the form of overviews, reviews, and assessments of research conducted in the two fields. The first author (PN) had the primary responsibility for finding and screening potential literature for the review. Literature identified as relevant was then read and discussed among all the authors until consensus on relevance for inclusion was reached. Literature that provided overviews of the research field, has been widely cited and is considered important within the field were prioritized. The following sources were used to provide an overview of policy implementation research: O’Toole Jr [8]; Schofield [9]; O’Toole Jr [10]; Barrett [11]; Schofield and Sausman [12]; Saetren [13]; Winter [7]; Sabatier [14]; Hill [15]; Hill and Hupe [16]; Paudel [17]; Johansson [18]; Cairney [19]; and John [20]. The following sources related to implementation science were used: Trinder and Reynolds [21]; Grol and Jones [22]; Greenhalgh *et al*. [23]; Grol *et al*. [24]; Estabrooks *et al*. [25]; Scott *et al*. [26]; Nutley *et al*. [27]; Estabrooks *et al*. [28]; Straus *et al*. [29]; Rycroft-Malone and Bucknall [30]; Brownson *et al*. [31]; and Grimshaw *et al*. [32].

## Discussion

### A brief overview of policy implementation research

Policy implementation research rose to prominence in the 1970s during a period of growing concern about the effectiveness of public policy [8,11]. The stage was set by Pressman and Wildavsky with the publication of their book entitled *Implementation* in 1973 [33]. Pressman and Wildavsky investigated the implementation of a federal economic development program to increase employment among ethnic minority groups in Oakland, California. From the beginning, policy implementation research was predominantly a North American enterprise.

Many first-generation studies were explorative, primarily seeking to position implementation within a policy cycle divided into a series of stages such as agenda setting, policy formulation, legitimation, implementation and evaluation. Implementation failure was described using a top-down approach, which identified factors to explain an implementation gap from the perspective of central government policy makers, *e*.*g*., unclear or flawed policy, insufficient resources, poor compliance by the implementers, opposition within the policy community, and unfavourable socioeconomic conditions [9]. The first generation of research has since been criticized for focusing too much on implementation failures (to the extent that it earned the nickname ‘misery research’ [34]) and, rather unfairly, for being ‘a theoretical’ and unable to produce convincing theories to help explain or predict the impact of policies [17]. Consequently, a second generation of studies emerged from the early 1980s with the ambition to take the next step in theory development by moving beyond a success or failure perspective towards improved analysis of variables that could explain the impact of the implementation process [9].

The construction of new analytical models and frameworks was accompanied by a debate between so-called top-down and bottom-up perspectives [19]. Bottom-up researchers critiqued the top-down perspective for viewing implementation as a purely administrative process and failing to account for the role of the frontline staff who put the policy into action [9]. Bottom-uppers shifted the analytical attention away from variables at the top or center of the system to the contextual and field variables at the bottom as the policy evolved in the complex process of translating policy intentions into action [10]. Lipsky [35] analyzed ‘street-level bureaucracy,’ focusing on the discretionary decisions that frontline staff make when delivering policies to citizens and organizations. He suggested that street-level bureaucrats could reasonably be described as policy makers.

Bottom-up researchers, many of whom were European, including Hanf and Scharpf [36], Hjern [37], and Hull and Hjern [38], focused their interest on the actions of the local implementers (and the importance of implementing structures or networks) as opposed to the central government, and emphasized not so much the goals of a policy but rather the nature of the social problem (*e*.*g*., youth unemployment or conditions for growth of small firms) that a policy was intended to address [7]. Bottom-uppers were less concerned with the implementation of a policy *per se* and more interested in understanding actor interaction in a specific policy sector [39]. Criticism directed at bottom-uppers included that they tended to overemphasize the autonomy of the frontline staff and lacked an explicit theory explaining what influenced the process and how change occurred [9]. The inductive nature of most bottom-up research combined with results that found most of the relevant factors varied from site to site led to few general conclusions or policy recommendations [40].

The top-down versus bottom-up debate had many facets, intertwining normative, methodological and theoretical issues [13]. Some top-down research exhibited a strong desire to develop generalizable policy advice, giving the research a prescriptive orientation [19]. Common top-down advice was to make policy goals clear and consistent [41], minimize the number of actors [33], limit the extent of change necessary [42], and place implementation responsibility with an agency sympathetic with the policy’s goals [39]. Bottom-uppers placed more emphasis on studying factors that caused difficulty in reaching stated goals. If the top-down perspective could be regarded as prescriptive, the bottom-up perspective focused on description of the implementation process. The bottom-uppers’ primary policy recommendation was for a flexible strategy that allows for adaptation to local difficulties and contextual factors [40]. The top-down and bottom-up perspectives were useful in drawing attention to the roles of the top and bottom of the implementation systems, but many in each camp ignored the portion of the reality explained by the other [7]. Convergence of the two perspectives was often deemed necessary for the field to develop, although Saetren [12:572] believes the ‘entrenched and prolonged debate frustrated many scholars to the extent that they exited the whole research enterprise’.

A third generation of policy implementation research emerged in the latter half of the 1980s, seeking to reconcile the two approaches by developing synthesized models and frameworks [19]. Several noteworthy models and frameworks emerged for improved understanding of implementation, including the Integrated Implementation Model [43], the Communication Model of Inter-Governmental Policy Implementation [44], and the Ambiguity-Conflict Model [40]. The importance of rigorous research methodology was emphasized, with more prominence given to longitudinal study designs and comparative multiple case studies to increase the number of observations [7,9,11].

Interest in policy implementation research seemed to stagnate in the 1990s, with decreased research activity and fewer publications in the core field. An important explanation for this decline was the changes that occurred in state–society relations in many industrialized countries, from unilateral and hierarchical to more reciprocal and horizontal relations. In the 1990s, there was more reliance on market-based policy instruments and less governmental intervention [13]. Policy implementation research shifted emphasis to address the effects of institutional and inter-organizational relationships, with governance and policy networks emerging as important research topics [8,13,16].

New terms developed to describe the same basic implementation processes associated with new forms of governing style [45]. The advent of the New Public Management led to the adoption of disciplinary approaches from management and organizational theory [12], some of which explore the extent to which top-down performance management could enhance service delivery and accountability [46]. Many governments subsequently recognized the limits to top-down policymaking and adopted network governance approaches based on the need to consult and collaborate with service providers, interest groups, and the users of services, blurring the lines of accountability between elected policymakers and other influential actors [47]. Bottom-up inspired governance studies highlighted the unintended consequences when governments did not recognise the limits to their ability to implement policy [48]. More recent studies, based on Complexity Theory, reinforce this focus on the limits to top-down policymaking in the alleged absence of central government control of the policy process [49].

The term implementation has become less popular but a focus on the same factors, such as the relationship between the production of policies and their effects at multiple levels of government, can still be found in a range of new fields. For example, the Advocacy Coalition Framework represents an attempt to reject a focus on implementation as a discrete stage of a policy cycle and, instead, theorizes the relationship between a huge number of governmental and non-governmental actors (driven by the desire to translate their beliefs into policy) at multiple levels, as policy changes over a decade or more [50,51]. Similarly, studies of multi-level governance try to capture that interaction between multiple actors at multiple levels, although the field is rather diverse and multi-level governance is, at best, an umbrella term [19].

Policy implementation studies can now be found at the intersection of public administration, organizational theory, public management research, and political science studies [12]. Publication of studies is predominantly seen in journals outside the traditional public administration field, suggesting that implementation research has become more multidisciplinary and diverse [7]. The emphasis is generally on domestic issues, with a bias towards the United States and Europe to a lesser degree. Global or international issues have received less attention [13], but there is growing interest in the study of European Union policies in member states and, in a small number of cases, the implementation of international agreements [52].

### Comparative analysis of implementation science and policy implementation research

This section provides a comparative analysis of the two fields, focusing on the following aspects: purpose and origins of the research; characteristics of the research; development and use of theory; determinants of change; and implementation impact.

### Purpose and origins of the research

Research on policy implementation and implementation science was borne out of a desire to understand, explain, and address problems associated with translating explicit and implicit intentions into desired changes. Both fields depict potentially damaging gaps between the expectations of the policy makers and the actual impact of the policy and between what research has shown to be effective and what is actually practiced in routine healthcare. It is generally assumed that research into implementation can generate knowledge to close or reduce these gaps.

Research on policy implementation emerged from the insight that political intentions seldom resulted in the planned changes, which encouraged researchers to investigate what occurred in the process and how it affected the results. The origins of implementation science can be traced to the emergence of evidence-based medicine and its wider application as evidence-based practice in the 1990s. These movements popularized the notion that research findings should be more widely implemented in various practice contexts [53]. The evidence-based movement has also influenced policymaking, yielding research on how research findings (*e*.*g*., assembled in systematic reviews) can be used to inform public policymaking [54]. Recognition that the rate of publication of new findings has become too large for healthcare practitioners to keep up to date has led to a stronger focus on research into strategies to facilitate research utilization and more evidence-based practice [55].

### Characteristics of the research

The policy implementation research that we review is generally part of social science. The study of policy implementation is a topic in public administration, which is a branch of political science, a field of research that deals with the theory and practice of politics and investigations into political systems and behavior. There have been calls for an overarching implementation theory, but policymaking is usually treated as too complex to attract a general theory. The case study method is commonly employed to account for a large number of causal factors [56]. Policy implementation research encompasses both more positivist approaches, as evident in some of the top-down research, and more interpretivist approaches, which are seen in many bottom-up studies that consider policies to be contestable and emergent in complex processes of interpretation and negotiation.

Policy implementation studies concern naturally occurring circumstances, meaning that the investigator is not able to control or manipulate different variables. The real-world circumstances and complexities of unpredictable policy processes that involve many actors pose significant methodological challenges to investigations. Studies in policy implementation research have used both qualitative and quantitative research methodologies, but there has been an emphasis on qualitative case studies [7,8]. Early research was dominated by single case studies, allowing implementation to be studied in a broad context. Case studies typically made use of several data sources, such as written documents and reports, interviews with implementers, and quantitative data concerning various aspects [17]. Third-generation research in the field sought to make greater use of multiple case studies and involve more longitudinal studies, to make the process ‘more scientific than the previous two’ generations [44:18]. However, it suffered from a lack of relevant data and an inability to distil a vast range of causes of policy outcome variation into a manageable and testable general theory [56].

Implementation science emerged in the wake of evidence-based medicine, initially showing a strong influence from medical research where the balance is tilted towards models of research practice drawn from the natural sciences. Early implementation science research tended to view the research–practice relationship as unidimensional and linear, with a flow of knowledge from the research community into the practice arena, *i*.*e*., a producer–push conceptualization of research use [27]. However, as evidence-based medicine has developed into evidence-based practice, research in implementation science has broadened and today incorporates theoretical and methodological approaches from social science even though it features far more quantitative research than seen in policy implementation research. Today, this research is also conducted from the perspective of the healthcare professionals’ perceptions and experiences, thus representing a sort of bottom-up perspective [57]. User–pull conceptualizations view research use as a process of learning in which healthcare professionals blend explicit research-based knowledge with implicit practice-based knowledge as they translate, adapt, and renegotiate research findings to make sense of them within the context of their everyday work [27].

Implementation science employs a variety of research methodologies, including the use of both observational and researcher-controlled experimental studies. The field’s cultural proximity to the evidence-based medicine/practice movement is evident in the research that involves testing the effectiveness of various strategies to achieve changes in clinical practice, preferably applying randomized controlled trial study designs, and systematic literature reviews to summarize the current knowledge of effective implementation interventions. Case studies are not afforded the same status as in policy implementation research. Qualitative research is most typically conducted to identify and describe problems in creating practice change and to generate hypotheses about determinants of change.

### Development and use of theory

In the policy implementation field, numerous models and frameworks that describe factors that may influence implementation endeavors have been suggested, including comprehensive checklists of large numbers of factors. However, these efforts have usually lacked explanation of the underlying causal mechanisms of the implementation process and have rarely addressed the relative importance of various independent variables [19]. The number of potential explanatory variables has been reduced over time, towards more parsimonious explanation [8]. However, much of the policy implementation literature can be said to feature thick description, whereby the implementation process is modelled or mapped out rather than explained with regard to causal mechanisms to provide the basis for a universal theory [19]. The goal of developing one overarching implementation theory has increasingly been called into question by researchers in the field. Johansson [18:117] notes that ‘nowadays, there seems to be no ambition to develop a general implementation theory’. Instead, leading researchers (*e*.*g*., [7,10,14,15] have argued that it is more fruitful to develop (and potentially test) different partial theories and hypotheses that address certain implementation aspects.

Implementation science researchers have made a conscious effort to import and use various theories that can improve the understanding, explanation, and prediction of implementation endeavors, irrespective of the origins or source of these theories. For example, Grol *et al*. [58] compiled a list of 15 types of theories that they advocated for use in the field, distinguishing between theories concerning individual healthcare practitioners (including social–cognitive theories on behavior change which are widely applied), theories related to social interaction and context, and organizational and economic theories.

Researchers in implementation science have also developed new field-specific theories, models, and frameworks. Many of these are specifically intended to help in planning implementation processes. Graham *et al*. [59] have referred to this category of theories as ‘planned action change theories,’ whereas existing theories (such as social–cognitive theories on behavior change) used in the field are labelled ‘classic change theories’. Examples of such action theories are the Promoting Action on Research Implementation in Health Services (PARIHS) model [60], the Iowa Model of Evidence-Based Practice [61], and the Knowledge-to-Action Framework [62]. It should be noted that the terminology is somewhat unclear, as these theories are also referred to as models and frameworks.

### Determinants of change

Determinants of change (also referred to as independent variables) are factors that are believed or have been found to affect the results of implementation endeavors. Whereas implementation science efforts [23,27,58,60,63-65] to describe independent factors typically encompass the same or similar core set of determinants, policy implementation efforts have been far more heterogeneous with regard to the number and classification of such factors due to the broader scope of the research field. Hence, it is important to emphasize that the framework applied below is a highly simplified representation of variables that may influence the implementation process and impact, primarily constructed for the comparative purposes of this study.

### Implementation object

The object that is implemented in implementation science can be seen as a specific clinical practice, *e*.*g*., ordering of laboratory tests, performing hand hygiene, or delivering health promotion advice, which has been found to be effective in research. The object in policy implementation research is a policy, *e*.*g*., a law or a regulation, developed by politicians and other policy makers. However, policy has a somewhat imprecise meaning; it may indicate an overall objective, a guiding principle, or a specific action that will be taken to reach an objective [66].

Although Pressman and Wildavsky [33] introduced the notion of a policy as an implementation object, definitions of policy as a specific phenomenon pose several difficulties. A policy may sometimes be identifiable in terms of a decision, but often involves a series of decisions or what may be seen as more of an orientation. Moreover, policies tend to change over time [15]. Defining an implementation object is further complicated by the fact that it is often difficult to determine a precise starting point of a policy because policy implementation typically presupposes prior activities in the form of agenda setting (deciding what problem to solve) and formulation (deciding how to solve it) [19,67]. Hence, policy implementation research faces problems in identifying what is being implemented because policies are complex phenomena. The object in implementation science tends to be a more easily identifiable and delimited phenomenon.

Implementation science research has established that certain features of the research findings and clinical guidelines are more likely to lead to adoption. Similarly, policy implementation researchers have suggested that the characteristics of a policy will affect its implementation [9]. Some researchers in this field have developed taxonomies of different types of policy, but they have generally refrained from specifying particular features of policies that are more favourable than others [15]. Furthermore, the complexity of the relationship between the process and impact of implementation precludes simple conclusions as to optimal policy characteristics [68,69].

### Implementers

The implementers are those who are responsible for implementing various implementation objects. In implementation science, the healthcare practitioners are usually considered the implementers. Individual practitioners ultimately decide whether or not to perform a specific clinical practice, such as prescribing an antibiotic for a sore throat, adhering to a hygiene recommendation, conducting a treatment follow-up, or providing advice on alcohol consumption. However, the practitioners do not exist in a vacuum, and their decisions are influenced by colleagues, managers, and many other factors that are considered part of the context in implementation science.

Policy implementation research describes implementers in terms of individuals and organizations, such as governmental authorities and public and private entities. Individuals (*e*.*g*., teachers, policemen, and physicians) who carry out the delivery of policies are referred to as frontline staff or street-level bureaucrats, whereas the organizations (*e*.*g*., authorities, schools, healthcare organizations) in which they work are often referred to as implementation agencies (or implementation entities). Contemporary perspectives on policy implementation take a holistic view of the implementers and describe complex networks of individuals, organizations, and inter-organizational relations, thus making it difficult to determine who the implementers are [16].

Implementation science research has established a number of characteristics of healthcare practitioners that are associated with greater research use and/or increased implementation of evidence-based practices. It is difficult to draw analogous conclusions about policy implementation due to the complexity of organizational processes involved in the policy process. Policy may be implemented by multiple actors at multiple levels; some control may be exerted from policy formation to the street level, but the lines of hierarchy may be unclear if the organizations that collaborate in the implementation endeavor are accountable to different policy makers [15].

### Targets

Targets are the individuals or organizations on which an implementation endeavor is ultimately intended to have an impact. Patients represent the targets in implementation science, whereas citizens and organizations are the targets in policy implementation research. Targets in policy implementation research are also referred to as clients and recipients.

Researchers in both fields acknowledge that the implementation process is influenced by the responses of those who are affected by what is implemented. This is evident in policy implementation research when the targets are powerful organizations or otherwise deemed more worthy of the attention of policy makers [70], but responses of weaker recipients such as clients of welfare programs may also influence the policy implementation process [16].

With regard to implementation science, features of the patients, such as their expectations, needs, attitudes, knowledge, and behavior, can have a strong influence on the care provided and achieving desired practice changes [65]. However, Grol *et al*. [58] note that there are no theories and only limited empirical research available to describe how or the extent to which clinical practice can be altered through the patient as a mediator.

### Context

The context is the social environment in which implementation takes place. Healthcare settings constitute the context in implementation science, whereas the context for policy implementation may be much larger. The context represents influences on the implementation process and impact that is, at least partially, beyond the control of the implementers and targets.

Implementation science typically describes contextual features as a determinant alongside others such as characteristics of the implementation object and the effectiveness of implementation strategies. It is customary in this field to distinguish between the inner context (or setting) and outer context (outer setting) of the implementation; the former represents features of the workplace or organization in which implementation takes place and the latter is related to the wider environment within which healthcare organizations reside [23]. The research–practice gap in implementation science has often been attributed to aspects of the inner context in terms of workplace or organizational characteristics such as time restrictions, limited access to research studies, and inadequate support from colleagues, managers, and other health practitioners [71,72].

Policy implementation research does not distinguish between the concepts of inner and outer context, but similar reasoning exists in this field. The inner context (in implementation science terminology) has been afforded great importance, particularly in bottom-up perspectives that address the relationships between the actors involved in the implementation process [11], *e*.*g*., between civil servants and their managers [35]. The outer context (in implementation science terminology) is typically understood to involve aspects of the policy environment in which policies are implemented, including demographic characteristics and global economic forces that might affect policy outcomes [16].

### Strategies to facilitate the implementation process

Implementation science describes various concerted strategies (also referred to as implementation interventions, facilitators, enablers, etc.) to influence the implementation process in order to achieve desired changes in clinical practice. The difference between strategies and implementers is not always clear. Implementation actors such as change agents, opinion leaders, or champions, as described in Rogers’ Diffusion of Innovation Theory [73], can influence the implementation process and be seen as both implementers and a strategy that facilitates the implementation process. Numerous taxonomies have been developed to categorize different strategies and assess their effectiveness; the Cochrane Effective Practice and Organization of Care framework is the most well-established [74]. Study findings concerning the effectiveness of various approaches are continuously synthesized and assembled in systematic reviews.

In policy implementation research, strategies to facilitate implementation are referred to as policy instruments (or government instruments). However, unlike implementation science, which isolates various strategies and distinguishes between the implementation object and strategies, policy implementation research considers strategies to be an integral part of the policy itself. However, different types of policy instruments have been categorized by several researchers in the field [19]. Although such taxonomies can make the research process more manageable, few policy implementation researchers have established, in the same way, what instruments might be most effective, an obvious reason being the inherent complexity of isolating this aspect of the implementation process [20].

### Implementation impact

Implementation processes are explicitly or implicitly aimed at achieving various types of changes among the implementers and targets. In implementation science studies, clinical behavior change and intentions to change clinical behavior are commonly used as dependent variables. Although some studies have investigated the extent to which evidence-based practices influence the targets (*i*.*e*., patients’ health), the extent to which practice change actually leads to the desired improvement among the patients has thus far received relatively limited research attention [75,76].

Policy implementation research, meanwhile, distinguishes between two types of dependent variables: output is the impact on the implementers (*i*.*e*., frontline staff and/or organizations involved in the implementation process) and outcome is the impact on the targets in society (*i*.*e*., citizens and organizations). Outputs are generally administrative decisions of some type (*e*.*g*., the decision to fund larger numbers of teachers, doctors, or police officers), whereas comparable outcomes include education attainment, improvements in health, and reductions in crime. Outcomes are often difficult to attribute directly to determinants and outputs.

Top-down approaches to policy implementation have predominantly studied implementation impact in terms of outputs or outcomes, usually investigating the degree to which policy goals have been attained [7]. Bottom-up and more negotiative approaches to implementation tend to view impact in terms of what is possible within a particular context, which groups have gained or lost, and how this has been affected by the policy [11]. The evaluative criteria can be anything the researcher decides is relevant to the study [39].

Measuring goal achievement outcomes poses several challenges that have led researchers such as Winter [7] and Hill and Hupe [16] to argue that more attention should be devoted to investigating output, *e*.*g*., in terms of behavioral variables that characterize the performance of implementers. Policy goals may be ambiguous, disputed, incompatible, and modified in the implementation process. Moreover, goals are not always expected or intended to be achieved. Further complicating the study of outcomes is the fact that these may be influenced by factors unrelated to the policy because outcomes are the results that are actually achieved, whether intended or unintended, regardless of what the goals of the policy might have been [15,19].

### Key differences between implementation science and policy implementation research

Despite limited crossover in the literature, there are many common issues in policy implementation research and implementation science. Research in both fields deals with the challenges of translating intentions into desired changes. While policy implementation is founded in social science, implementation science has adopted many principles from the evidence-based medicine and evidence-based practice movements drawn from the natural sciences. Still, both fields emphasize the importance of interdisciplinary research using a variety of research methodologies in this enquiry.

However, there is a fundamental difference in the potential complexity of the phenomena under study in the two fields. The implementation object in policy implementation research (albeit a contested concept) ranges from the relatively concrete and easily defined (*e*.*g*., regulation of smoking in public places or changes in sickness insurance regulations) to broader and longer-term policy development such as the influence of political coalitions on political development over decades. Furthermore, the implementation process often involves many interdependent actors, sometimes spanning many years. Sabatier [14:3] believes the process concerns ‘an extremely complex set of elements that interact over time’. Similarly, John [20:7] argues that this process is ‘hard to research effectively as it is composite of different processes that crosscut most branches of government and involve many decision makers’. Furthermore, the outputs and outcomes of the implementation endeavor can be very heterogeneous, *e*.*g*., the number of older people receiving adequate personal care, levels of student debt, or the amount of time that people wait for medical treatment. In contrast, implementation science focuses on specific clinical practices described in research and their adoption in a relatively short time perspective by healthcare practitioners in healthcare settings.

The narrower scope of implementation science has allowed for a more reductionist approach to the study of implementation. Implementation science researchers have distinguished between a number of individual determinants that are causally linked with outputs and outcomes, and considerable research effort has been devoted to investigating the effectiveness of specific strategies to affect these results. The strong influence from the medical sciences can be seen when researchers in the field [77,78] compare the linking of strategies to overcome implementation barriers to the tailoring of clinical treatments to diagnosed health problems. Policy implementation researchers have to a greater extent stressed the inherent interdependency between various factors as well as the crucial importance of the context, which makes it difficult to generalize findings on the relative importance of individual determinants. The fit between different factors, *e*.*g*., policy characteristics and the strategies undertaken to implement a policy, is generally considered to be more important than the characteristics or strategies themselves.

There are some similarities concerning the view of the implementation process in the two fields. Thus, the top-down perspective on policy implementation research is very much echoed in early implementation science research that was premised on a rational sequential model of the implementation process. However, implementation science research has evolved and is also conducted from the perspective of healthcare professionals’ perceptions and experiences, an approach that is clearly reminiscent of the bottom-up perspective on policy implementation. Top-down perspectives on the implementation process imply a more positivist orientation as the implementation object is often viewed in terms of an entity that exists in a finished form as explicit objective facts (*e*.*g*., recommendations for certain clinical practices formulated in guidelines) and the implementation process is considered primarily as an act of ‘transporting’ this knowledge to potential users. Bottom-up perspectives suggest a more interpretivist understanding of the process, as the implementation object is interpreted, subjective, and contestable. Implementation science researchers tend to acknowledge the relevance of both approaches, and prominent researchers in this field conduct many different types of studies. Hence, implementation science has not experienced a polarization of standpoints and the type of ‘protracted and sterile debate’ [12:572] seen in policy implementation research has been avoided.

Whereas some earlier policy implementation researchers have had the ambition to develop a general implementation theory, there have been few calls for an overarching theory in implementation science. The theory closest to achieving the status of an all-encompassing theory in this field is Rogers’ Diffusion of Innovation Theory, developed in the 1950s in rural sociology, which seeks to explain the spread of new ideas [73]. The theory continues to be widely used in many fields, including implementation science.

Researchers in both fields have developed numerous field-specific models and frameworks. However, implementation science researchers have also pragmatically looked to other fields and disciplines to borrow theories, models and frameworks. Policy implementation researchers seem to have been more cautious about using theories derived in other fields. Some researchers in the field [15] have warned against uncritical combination of theories that may be based on different assumptions, although there are also researchers [9,14] who advocate increased use of exogenous theories in policy implementation research.

### Learning from policy implementation research

Bearing in mind these differences between the two fields, the question arises whether there are lessons from policy implementation research that might have bearing on implementation science. We believe there are several aspects of policy implementation and related research that have relevance for the implementation science field. Policy implementation researchers, particularly the bottom-uppers, have focused a great deal of attention on the context of implementation. In general, the context appears to be a less understood mediator of change in implementation science. It has been argued that a more sophisticated and active notion of the context is needed than is displayed in much of the existing implementation science literature [79]. However, there is growing interest in theories concerning organizational culture, organizational climate, and leadership, as well as concepts and constructs such as absorptive capacity and readiness for change [80,81].

The bottom-up policy implementation perspective has shown the importance of understanding the rules, values, and norms of the implementers, as well as recognizing that the influence of new knowledge must be considered alongside the enduring effect of the implementers’ long-standing practices. The street-level bureaucrats’ decisional latitude, as depicted by Lipsky [35], can clearly be seen as analogous to the discretion of healthcare practitioners to choose the knowledge on which to act. In both cases, practitioners may not implement all of the top-down recommendations, instead using their discretion to establish routines to satisfy a proportion of government objectives while preserving a sense of professional autonomy.

These findings point to the relevance of exploring how healthcare practitioners influence the implementation process by being part of professional subcultures, communities of practices, and social networks that affect the spread of ideas, knowledge, and learning in healthcare [82-84]. Implementation science studies tend to focus on various individual attributes of healthcare practitioners (*e*.*g*., their decision-making, knowledge, skills, and attitudes) but group and aggregate levels might be equally or more relevant analytical units to understand practice changes in healthcare.

The Advocacy Coalition Framework [14] also examines the potential to adopt new policy ideas and the ways in which ideas are interpreted and policies adopted. Different stakeholders, often with very different beliefs about how the world works and what constitutes good evidence, shape what is seen as socially valid knowledge or the practical meaning of ‘evidence,’ which may influence the implementation of policies and guidelines. Thus, the implementation and widespread use of certain treatments (such as cognitive behavioral therapy) or organizational concepts (such as Lean production) may take place through the lens of a belief system that differs markedly from the belief system used to generate the initial research.

Policy implementation research has recognized the importance of the wider policy environment. Many studies in this field have struggled with the difficulties of separating the effects of this outer context from the specific effects of policies [56]. In comparison, the influence or characteristics of the outer context, including the political and cultural milieu in which healthcare is carried out, appears to be considerably less recognized in implementation science. Indeed, the outer context is not always included in frameworks that categorize implementation determinants in this field (*e*.*g*., [65,81,85]). In comparison with the inner context, potential outer context determinants are not as clearly manifested and may be difficult to identify with certainty, thus making it difficult to establish how they influence the implementation endeavor. However, policy implementation research has shown that the outer context is not a passive backdrop, but is actively brought into implementation processes and may cause unintended outcomes.

Policy implementation research has dealt with both outputs (changes among the implementers) and outcomes (changes among those targeted with the policy). Policy research that views the policy process as co-production, involving negotiation and bargaining with the targets [16], points to a relevant area of investigation for implementation science: how do and can patients influence the care provided and affect practice changes in healthcare? Implementation science has focused very much on outputs rather than outcomes, *i*.*e*., on healthcare practitioners rather than patients. It is implicitly assumed that the use of research-based knowledge will yield beneficial outcomes for patients, as demonstrated in the research on which various practices are based. However, critics of the evidence-based movement have complained that there is insufficient empirical evidence of the effectiveness of implementing a more evidence-based healthcare practice [86]. However, an increasing number of recent studies have shown positive links between evidence-based practices and patient health, *e*.*g*., concerning the association between adherence to clinical guidelines and patient outcomes [87-89].

Although the use of the term policy implementation term has decreased, research on implementation and/or policy issues has continued under other labels within political science. Governance Theory lessons can be drawn on although the literature is more difficult to pin down (as is the meaning of governance) and less focused on implementation. This research has shown the relevance of investigating how top-down implementation measures can cause unintended consequences [48]. For example, the use of targets in one area, such as reduced waiting times in healthcare or improved patient safety performance, may produce disproportionate resource allocation to achieve short-term results at the expense of the longer-term results associated with the use of research findings in healthcare [90].

Institutional Theory offers further explanation for the difficulties with implementing research-based knowledge and achieving desired practice changes in healthcare. A central argument of this perspective is that the adoption and use of new practices is not solely a means of improving performance, but as much a process of achieving legitimacy within a certain social context. The institutional perspective assumes that conditions of uncertainty in relation to environmental forces and goals lead to organizations imitating other organizations [91,92]. The decision to adopt new practices might therefore relate more to institutional pressures associated with various fads and fashions than to well-founded evidence to support their use. By emphasizing intra- and inter-organizational processes, an institutional perspective may thus contribute to improved understanding of factors beyond the realm of evidence, research, or professional development that influence practice changes in healthcare.

There is also research and knowledge in other disciplines of public administration that might be valuable in the study of various healthcare implementation problems, *e*.*g*., issues concerning resource allocation, priorities, and the distribution of power between different stakeholders such as politicians, administrators, healthcare professional groups, and patient groups. Furthermore, knowledge on topics such as knowledge management, organizational behavior, and professional roles and power might contribute to better understanding and explanation of complex implementation processes in healthcare.

This narrative review has important limitations that must be considered. We relied on a limited number of sources and did not set out to provide a comprehensive review of the two fields. We compared a few aspects of the two fields. Still, it is obvious that there are profound differences that must be acknowledged and which provide an explanation for the limited knowledge exchange between the fields. The lack of collaboration is not due to a paradigm war or even a debate about the relevance of different perspectives; it is more a question of mutual ignorance.

We believe there is important learning for implementation science researchers to be derived from several aspects of policy implementation research and from associated research into various implementation and/or policy issues in political science. Implementation science is at a relatively early stage of development, and advancement of the field would benefit from accounting for knowledge beyond the parameters of the immediate implementation science literature. We agree with the noted public policy researcher O’Toole [7:283], who believes that ‘it behooves scholars not to draw arbitrarily narrow jurisdictional lines, nor to expend energy on sectarian causes.’ Ultimately, a broad, multidisciplinary research enterprise is needed to realize the ambitions of improved implementation of research findings in healthcare and achieving a more research-informed clinical practice.

## Summary

There are many common issues in policy implementation research and implementation science. Research in both fields deals with the challenges of translating intentions into desired changes. However, while policy implementation is founded in social science, implementation science has adopted many principles from the evidence-based medicine and evidence-based practice movements drawn from the natural sciences. Still, both fields emphasize the importance of interdisciplinary research using a variety of research methodologies in this enquiry. Researchers in both fields have developed numerous field-specific models and frameworks, but implementation science researchers have also pragmatically looked to other fields and disciplines to borrow theories, models and frameworks. Policy implementation researchers appear to have been more cautious about using theories derived in other fields.

Implementation science researchers have distinguished between a number of individual determinants that are causally linked with outputs and outcomes, and considerable research effort has been devoted to investigating the effectiveness of specific strategies to affect these results. Policy implementation researchers have to a greater extent stressed the inherent interdependency between various factors as well as the crucial importance of the context, which makes it difficult to generalize findings on the relative importance of individual determinants. Policy implementation research has dealt with both outputs (changes among the implementers) and outcomes (changes among those targeted with the policy). In contrast, implementation science has focused very much on outputs rather than outcomes, *i*.*e*, on healthcare practitioners rather than patients.

Important learning may be derived from several aspects of policy implementation research, including issues related to the influence of the context of implementation and the values and norms of the implementers (the healthcare practitioners) on implementation processes. Relevant research on various associated policy topics, including The Advocacy Coalition Framework, Governance Theory, and Institutional Theory, may also contribute to improved understanding of the difficulties of implementing evidence in healthcare.

## Competing interests

The authors declare that they have no competing interests.

## Authors’ contributions

The idea for the paper was conceived by PN, CS and KR. The first version of the article was drafted by PN. It was revised, with contributions made by all other authors, CS, KR and PC. All four authors then jointly assembled and revised further versions. All authors read and approved the final manuscript.
